# A sensitive multidimensional method for the detection, characterization, and quantification of trace free drug species in antibody-drug conjugate samples using mass spectral detection

**DOI:** 10.1080/19420862.2015.1116659

**Published:** 2015-12-14

**Authors:** Robert E. Birdsall, Sean M. McCarthy, Marie Claire Janin-Bussat, Michel Perez, Jean-François Haeuw, Weibin Chen, Alain Beck

**Affiliations:** a Waters Corporation, 34 Maple Street, Milford, MA, 01757, USA; b IRPF, Center d'Immunologie Pierre Fabre, St Julien-en-Genevois, France; c IRPF, Center de R&D Pierre Fabre, Toulouse, France

**Keywords:** ADC, AFC, antibody-drug conjugate, antibody-fluorophore conjugates, drug mimic, free drug, multidimensional chromatography, maleimide-linker-drug mimic, NAc-linker mimic, SPE/RPLC/MS, solid phase extraction, 2DLC

## Abstract

Conjugation processes and stability studies associated with the production and shelf life of antibody-drug conjugates (ADCs) can result in free (non-conjugated) drug species. These free drug species can increase the risk to patients and reduce the efficacy of the ADC. Despite stringent purification steps, trace levels of free drug species may be present in formulated ADCs, reducing the therapeutic window. The reduction of sample preparation steps through the incorporation of multidimensional techniques has afforded analysts more efficient methods to assess trace drug species. Multidimensional methods coupling size-exclusion and reversed phase liquid chromatography with ultra-violet detection (SEC-RPLC/UV) have been reported, but offer limited sensitivity and can limit method optimization. The current study addresses these challenges with a multidimensional method that is specific, sensitive, and enables method control in both dimensions via coupling of an on-line solid phase extraction column to RPLC with mass spectral detection (SPE-RPLC/MS). The proposed method was evaluated using an antibody-fluorophore conjugate (AFC) as an ADC surrogate to brentuximab vedotin and its associated parent maleimide-val-cit-DSEA payload and the derived N-acetylcysteine adduct formed during the conjugation process. Assay sensitivity was found to be 2 orders more sensitive using MS detection in comparison to UV-based detection with a nominal limit of quantitation of 0.30 ng/mL (1.5 pg on-column). Free-drug species were present in an unadulterated ADC surrogate sample at concentrations below 7 ng/mL, levels not detectable by UV alone. The proposed SPE-RPLC/MS method provides a high degree of specificity and sensitivity in the assessment of trace free drug species and offers improved control over each dimension, enabling straightforward integration into existing or novel workflows.

## Abbreviations

ADC, antibody-drug conjugate: 1D, 1^st^ dimension
2D, 2^nd^ dimension BSM, binary solvent manager
CQA, critical quality attribute: DAR, drug-antibody-ratio
DSEA, dansyl sulfonamide ethyl amine: ELISA, enzyme linked immunosorbent assay
LC-UV, liquid chromatography ultraviolet LOQ, limit of quantitation
mAb, monoclonal antibody: Mal, maleimidocaproyl
MP, mobile phase: MS, mass spectrometry
NAc, N-acetyl-cysteine: QSM, quaternary solvent manager
QTOF, quadrupole time-of-flight: RP, reversed phase
CE-SDS-CE, sodium dodecyl sulfate-capillary electrophoresis: SEC, size exclusion chromatography
SNR, signal-to-noise ratio SPE, solid phase extraction
TCEP, (tris(2-carboxyethyl)phosphine): TUV, tunable ultra-violet; UV, ultraviolet.

## Introduction

Antibody-drug conjugates represent a growing class of immunoconjugate therapies for the treatment of cancer.^1-4^ Cytotoxic agents based on auristatin^5^ and maytansines^6^ are too potent to be used in traditional cancer treatment strategies such as chemotherapy. To overcome this challenge, highly potent drugs such as these are covalently attached to a linker molecule and conjugated to a monoclonal antibody (mAb) via cysteine residues associated with engineered sites,^7,8^ unnatural amino acids,^9,10^ or reduction of inter-chain disulfide bonds^11^ as in the case of brentuximab vedotin (Adcetris®)^12^ or through primary amines associated with lysine residues such as in ado-trastuzumab emtansine (Kadcyla®).^13^ The conjugation of potent drugs to a mAb enables the targeted delivery of toxic payloads to tumor surfaces while minimizing systemic toxicity effects to healthy tissue, thus improving the therapeutic window for such modalities in the treatment of cancer.^7,14^ Incomplete conjugation processes can result in free or non-conjugated drug, drug-linker, or drug-related impurities that co-exist with the ADC molecules in the samples. Additionally, degradation products can occur over time in formulation as well as in vivo circulation, increasing the risk to patients and reducing the efficacy of the ADC.^15-17^ Trace levels of these free drug species may still remain in formulated ADCs despite the inclusion of multi-purification steps during the production process. For these reasons, characterization and quantification of residual free drug and associated products is required to ensure a safe and efficacious product.

Currently, common methods for the detection of trace-level free drug species include enzyme-linked immunosorbent assays (ELISA),^16,18,19^ reversed phase liquid chromatography (RPLC) techniques,^20,21^ and solid-phase extraction (SPE).^22^ The merits of these methods in the assessment of residual free drug species are well established, but are not without their challenges. ELISA-based assays offer a high degree of specificity, sensitivity, and throughput. However, ELISA assays suffer from lengthy assay development time, cross reactivity with related drug impurities, lack of specificity toward degradation products, and reduced binding efficiency due to matrix effects.^19,23,24^ RPLC techniques are well established in the analysis of small molecules associated with pharmaceuticals, but direct injection of ADC samples onto a RP column without any prior sample treatment can lead to column fouling, detection interferences, as well as carry-over. Thus the use of RPLC for trace free drug analysis typically requires additional sample preparation and column conditioning steps.^25,26^ Sample preparation methods to remove protein species have included precipitation techniques that involve diluting samples with organic solvents to precipitate protein components allowing for removal of hydrophobic free drug species with the supernatant^27,28^ or SPE techniques employed in either off-line or on-line formats to extract small molecule analytes, such as drug species, from various matrices for further analysis.^22,29^ While effective to remove interfering matrices, these approaches require additional steps to concentrate samples prior to analysis and can lead to loss of free drug through non-specific interactions.^30-32^ As potentially more potent drug candidates for ADCs are identified, efforts to expand the therapeutic window will require assays with improved sensitivity for the assessment and characterization of residual free drug species to ensure product safety and efficacy.^33,34^

Recently, multidimensional chromatography has been reported in the characterization of drug products,^35,36^ production excipients,^37^ and surfactants^38^ associated with biopharmaceuticals as well as other areas.^39–41^ These application examples have demonstrated the potential of such techniques for the characterization of complex samples such as ADCs. Work by Fleming *et al.* successfully coupled a commercially available mixed mode column (1^st^ dimension) with a RP separation in the 2^nd^ dimension to study accelerated degradation products of maytansinoid-conjugated antibodies.^35^ While the method was successful in assessing both relative and absolute abundance of degradation products, the use of ultra-violet (UV) detection offered a rather limited response for the detection of trace level drug degradation products. In another study, He and colleagues recently coupled SEC with a mixed mode stationary phase for the assessment of multiple production excipients in a panel of large molecule types.^37^ Although their work demonstrated the utility of multi-attribute monitoring of complex samples using multidimensional chromatography, the potential of the methodology for assessing trace-level free drug species associated with ADCs was not addressed. More recently, Li *et al.* successfully coupled size-exclusion chromatography (SEC) to RPLC for stability studies of drug molecules related to ADCs.^36^ Their study successfully increased the sensitivity of the analysis by 10-fold when UV absorption was used as the detection method. The increased detection performance was achieved through the implementation of 2 dimensions, bypassing off-line sample preparation steps. Although mass spectral (MS) analysis was in-line in the system configuration, it was used primarily for identification of degradation products rather than as a means to quantify the free drug.

The utility of MS in the characterization of ADCs has been well established in literature. Work by Lazar *et al.,**^42^* Valliere-Douglass *et al.,**^43-45^* and others^46-50^ have successfully applied native or denatured MS-based techniques in the characterization of critical quality attributes (CQAs) such as drug-antibody-ratio (DAR), drug distribution, and conjugation site identification. Multidimensional chromatography approaches, when coupled to MS analysis, have been shown to improve sensitivity by reducing ion suppression caused by matrix effects and salt-adducts.^41,51,52^ Coupling 2 orthogonal dimensions in a single method provides the ability to bypass lengthy sample preparation steps,^36^ hyphenate otherwise non-compatible techniques (e.g., couple a separation using non-volatile buffers with MS analysis),^41^ and enhance separation efficiency;^53^ attributes that are desirable in the characterization techniques for CQAs of ADCs in formulation or biological matrices. SEC-RPLC methods, when used in free-drug characterization, lack a high degree of flexibility in method optimization for the first dimension separation by SEC - a potential dilemma considering the diversity of biological substrates and drug candidates currently under investigation can exhibit varying physicochemical properties that affect chromatographic performance. Furthermore, UV-based detection methods, while adaptable, offer limited sensitivity for detecting free drug and its analogs at trace levels, thus hindering the effort to improve the therapeutic window of ADCs. Collectively, a single method that can be rapidly deployed with minimal sample preparation, yet offer the specificity and sensitivity for detection of trace level free drug species, is highly desirable.

We developed an integrated approach to address these challenges through the targeted extraction of free hydrophobic drug species using an online mixed mode SPE column. The unique selectivity offered by mixed mode chemistries has been successfully used in the extraction of APIs, metabolites, and detergents from a variety of matrices.^22,29^ Extraction of analytes of interest is accomplished through the exploitation of orthogonal physicochemical properties such as charge and hydrophobicity. Conceptually, molecules such as ADCs, will not be adsorbed on the SPE column because both the ADC molecules and sorbent surface (a hydrophobic polymer chain interspersed with amine functional groups) bear the same net positive charge under acidic conditions. The non-conjugated or free drug species bearing a net neutral or basic (negative) charge are adsorbed to the hydrophobic polymer backbone of the SPE ligand, and enriched for downstream analysis.^29^ The selectivity of the SPE column toward free drug species when coupled to RPLC/MS analysis, facilitates a means for analysts to tune the specificity of the method for a diverse group of substrates or drug candidates while maintaining sensitivity in an efficient on-line format. The objective of this study is to demonstrate a multidimensional approach with mass detection for improved specificity and sensitivity in the detection, characterization, and quantification of trace free drug species in unadulterated ADC samples enabling straightforward integration into existing or novel workflows.

## Results

**Figure 1.**
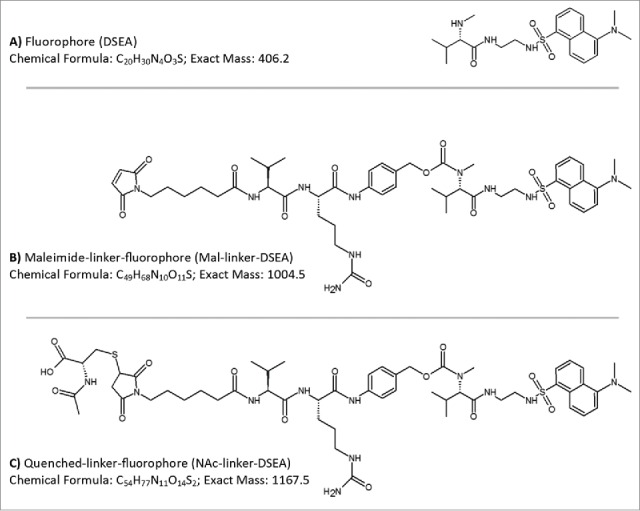
AFC mimic drug components. Drug components used in the production of a non-toxic AFC to mimic chemistry and linker species of brentuximab vedotin were based on a (**A**) dansyl sulfonamide ethyl amine (DSEA) moiety attached to (**B**) a maleimidocaproyl valine-citrulline linker species (Mal-linker-DSEA). Residual reactive mal-linker-DSEA was quenched with N-acetyl-cysteine following the conjugation step, producing a (**C**) quenched-linker-fluorophore (NAc-linker-DSEA) adduct species.

To test the broadest applicability of the proposed method for free drug analysis in ADC samples, selected molecules should possesses the key structural features of a typical ADC (e.g.,, common conjugation methods and linker structures) and preferably exhibit low cytotoxicity for ease of use and handling. Substitution of mono­methylauristatin E (MMAE) with a dansyl sulfonamide amine (DSEA, Fig. 1A) while maintaining the maleimidocaproyl valine-citrulline linker (mal-linker-DSEA, Fig. 1B), which is actively used in ADCs, successfully meets these criteria.^47^ The antibody-fluorophore-conjugate (AFC) mimics physiochemical characteristics of the clinically relevant ADC brentuximab vedotin (Adcetris®),^12,54^ and has minimal cytotoxicity due to the use of a non-cytotoxic drug mimic. An in-depth study of the manufactured AFC carried out by Wagner-Rousset and colleagues demonstrated the integrity of the ADC surrogate was not compromised and was well suited for research and development of ADCs.^47^ As part of the conjugation process, reactive maleimide containing a valine-citrulline linker that did not undergo conjugation were quenched through the addition of N-acetyl-cysteine (NAc-linker-DSEA, Fig. 1C) and removed via SEC purification. Assessment of residual drug species was carried out using a multidimensional chromatography method that couples an online SPE mixed mode anion exchange column (Oasis MAX, Waters) with a superficially porous high resolution C_18_ column (Cortecs C_18_, Waters) with in-line detection performed simultaneously using a tunable UV (ACQUITY TUV, Waters) and single quadrupole MS detector (ACQUITY QDa, Waters).

### Reference standards

**Figure 2.**
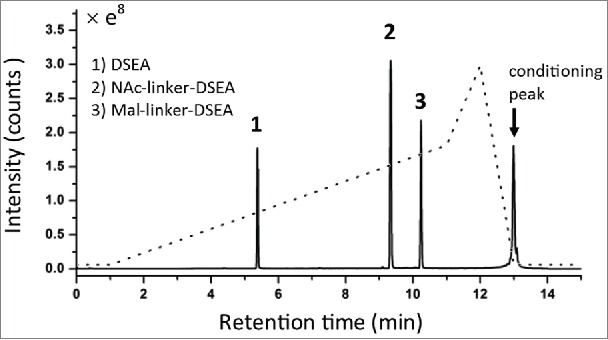
Reference standard evaluation. DSEA, NAc-linker-DSEA, and Mal-linker-DSEA reference standards were separated over a 10 min gradient from 5 % - 50 % (dashed line) with acetonitrile containing 0.1 % FA v/v, as the organic mobile phase using a superficially porous C_18_ RPLC column. Combined spectrum from SIRs collected using the [M+1H]^+1^ and [M+2H]^+2^ charge state for each component using optimized MS settings (see experimental) is shown.

Reference standards composed of DSEA, linker-DSEA, and NAc-linker-DSEA were analyzed using an ACQUITY H-Class Bio with 2D technology (Waters Corp.) in a 1DLC configuration. A 10 min reversed phase (RP) gradient separation was performed using a superficially porous 2.1 × 50 mm, 2.7 um C_18_ column (Cortecs C_18_, Waters) to assess the suitability of the reference standards for the proposed method. Selected ion recording (SIR) of the [M+1H]^+1^ and [M+2H]^+2^ ions were collected for each standard. As shown in Fig. 2, a mixture of the 3 reference standards were base-line resolved within the applied gradient. Interestingly, the DSEA drug mimic was notably less hydrophobic than the mal-linker-DSEA and NAc-linker-DSEA standards, eluting at 24% organic mobile phase (MP) composition compared to 42% and 46% MP composition for the NAc-linker-DSEA and mal-linker-DSEA standards, respectively. Relative retention times of the NAc-linker-DSEA and mal-linker-DSEA agree with RPLC separation results observed by Li *et al.**^36^* using the same valine-citrulline linker. The free drug mimic (DSEA) was less hydrophobic in comparison to the ADC warhead in Li's study and may be less diagnostically relevant for the current study. Choice of ion pairing agent, column selection, and possible increased hydrophobicity of the active pharmaceutical ingredient (API) of the warhead used in the study performed by Li *et al.* prevents a direct comparison to the current study. Nonetheless, the valine-citrulline linker and its N-acetyl-cysteine adduct, which exhibit similar RP characteristics as observed by Li *et al.**^36^* and are commonly used in auristatin-based ADCs, were determined to be fit-for-purpose for the current study to assess the proposed multidimensional method and by extension will be predictive of API warheads that exhibit similar hydrophobic characteristics.

### Reference standard calibration plot

**Figure 3.**
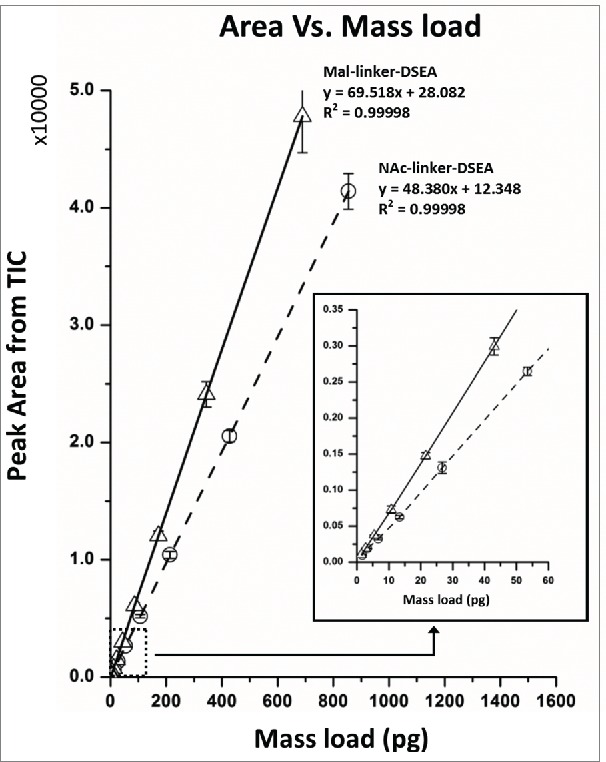
Assay dynamic range. Analysis of reference standards were performed in triplicate. Calibration plots of the reference standards were generated using peak area from SIRs for the most abundant [M+2H]^+2^ charge state and fitted with an ordinary linear regression model. Using ICH guidelines the MS quadrupole dynamic range was determined to be 1.35 pg – 688.5 pg for the mal-linker-DSEA and 1.65 pg – 854.5 pg for the NAc-Linker-DSEA reference standards.

Serial dilutions of the mal-linker-DSEA and NAc-linker-DSEA standards were analyzed in triplicate using a 10 min RP gradient with the same instrument settings and column configuration used in Fig. 2. Data acquisition was performed in tandem using a tunable UV detector set at an absorbance wavelength of 280 nm and a single quadrupole MS detector using SIRs for the most abundant charge state for each standard which was observed to be the [M+2H]^+2^ ion (> 97 % relative abundance). The total ion chromatogram (TIC) for the SIR associated with each drug component were integrated to determine peak area. A plot of peak area versus on-column analyte mass load was examined using regression analysis to determine the linearity of the MS detector response (Fig. 3) and TUV chromatograms (data not shown).^55^ Assay suitability was evaluated using regulatory guidelines^56^ for precision (< 20% R.SD at the limit of quantitation (LOQ), otherwise <15%) and accuracy (< 20% relative error (R.E.) at the LOQ, otherwise <15%) to determine the dynamic range of the method.

An ordinary linear regression was determined to model the data accurately. The dynamic range (Table 1) for the single quadrupole was determined to span 2.5 orders of magnitude (0.27 ng/mL – 137.70 ng/mL) for the mal-linker-DSEA standard and 2.5 orders of magnitude (0.33 ng/mL – 170.90 ng/mL) for the NAc-linker-DSEA standard with LOQs of 0.27 ng/mL (1.35 pg on-column; SNR = 9.56) and 0.33 ng/mL (1.65 pg on-column; SNR = 9.97), respectively. The dynamic range results combined with the good agreement of fitted data at lower concentrations (Fig. 3, inset), suggests a limit of detection of 0.10 ng/mL (0.5 pg on-column) should be achievable with the proposed assay. In practice, an undesirable level of data processing is required at lower concentrations for proper integration, preventing accurate analysis. Nonetheless, the ability to detect target compounds at a nominal 0.3 ng/mL (1.5 pg on-column) demonstrates the methods ability to detect drug compounds with a high degree of sensitivity.

**Table 1.** Assay suitability. Analyses of standards were performed in triplicate and evaluated using ICH guidelines for precision (< 20 % R.SD at the LOQ, otherwise <15 %) and accuracy (< 20 % relative error (R.E.) at the LOQ, otherwise <15 %). The dynamic range was extended 2 orders of magnitude using the quadrupole MS detector in a serial configuration with the LC-TUV optical detector.

Mal-Linker-DSEA								
**N=3**			**TUV**			**MS**		
Ref. Sample	Conc. (ng/mL)	Mass load (pg)	Area	R.SD	R.E.	Area	R.SD	R.E.
				(%)	(%)		(%)	(%)
1	4406.25	22031.25	196.67	2.35	99.80	1195858	18.32	78.08
2	2203.13	11015.65	99.35	2.13	100.55	667774	11.20	87.20
3	1101.56	5507.80	50.05	1.80	100.74	358237	9.78	93.55
4	550.78	2753.90	25.53	1.75	101.66	184526	8.42	96.37
5	275.39	1376.95	12.80	3.56	99.71	94516	7.52	98.71
6	137.70	688.50	6.64	4.04	99.25	47794	6.48	99.80
7	68.85	344.25	3.20	2.14	88.35	24108	4.49	100.62
8	34.42	172.10	1.67	2.73	80.16	12057	3.17	100.53
9	17.21	86.05				6089	4.47	101.29
10	8.61	43.05				2992	4.00	99.08
11	4.30	21.50				1476	2.95	96.89
12	2.15	10.75				728	6.81	93.78
13	1.08	5.40				373	0.61	92.79
14	0.54	2.70				191	2.61	88.67
15	0.27	1.35				103	2.74	85.11
**NAc-Linker-DSEA**								
**N=3**			**TUV**			**MS**		
**Ref. Sample**	**Conc. (ng/mL)**	**Mass load (pg)**	**Area**	**R.SD**	**R.E.**	**Area**	**R.SD**	**R.E.**
				(%)	(%)		(%)	(%)
1	5468.75	27343.75	262.41	1.23	100.17	1081872	18.77	81.78
2	2734.38	13671.90	131.17	2.07	99.31	591076	14.83	89.36
3	1367.19	6835.95	66.52	2.35	99.08	311053	10.56	94.05
4	683.59	3417.95	35.87	3.50	103.49	161949	6.75	97.93
5	341.80	1709.00	18.59	4.21	100.90	82233	4.81	99.44
6	170.90	854.50	10.66	2.06	103.42	41406	3.66	100.13
7	85.45	427.25	5.61	2.62	89.68	20543	2.41	99.32
8	42.72	213.60				10409	2.77	100.60
9	21.36	106.80				5178	2.99	99.96
10	10.68	53.40				2646	2.15	101.93
11	5.34	26.70				1311	6.12	100.54
12	2.67	13.35				625	3.28	94.91
13	1.34	6.70				322	4.18	95.89
14	0.67	3.35				190	2.45	109.53
15	0.33	1.65				95	5.33	102.17

The linear dynamic range of the TUV measurement (Table 1) was determined to span 2 order of magnitude for mal-linker-DSEA (34.42 ng/mL – 4,406.25 ng/mL) and NAc-linker-DSEA (85.45 ng/mL – 5,468.75 ng/mL) standard with the LOQ determined to be 34 ng/mL (0.17 ng on-column) and 85 ng/mL (0.43 ng on-column), respectively. The UV measurement was not evaluated at higher concentrations to extend the dynamic range as higher concentrations would be outside current regulatory recommendations for allowable impurity levels. The lower sensitivity (or the higher detection limit) of the TUV measurement was not unexpected, and supports the investigation to configure in-line MS detection for improved sensitivity. Incorporation of mass detection extended the sensitivity of the proposed method 2 orders of magnitude beyond traditional UV-based detection and was 150-fold more sensitive with an LOQ of 0.30 ng/mL (1.5 pg on-column) for free-drug species compared to previously published methods that used a SEC-RPLC/UV configuration for a similar compound.^36^

### Optimization of solid phase extraction

**Figure 4.**
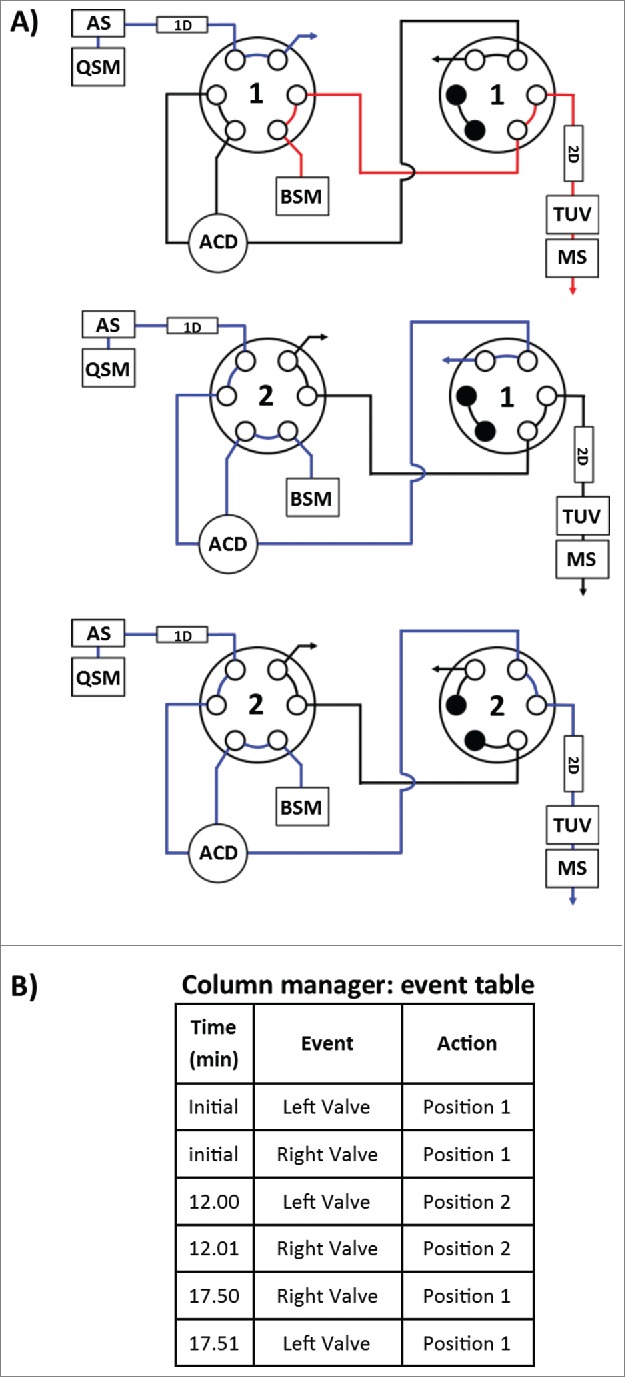
Instrument configuration schematic. (**A**) A column manager housing 2 6-port 2-position valves was configured as illustrated by the schematic to facilitate transfer of retained drug species between the SPE (1^st^) and RPLC (2^nd^) dimensions. Valve position is denoted numerically as position 1 and 2. Abbreviations are defined as QSM: quaternary solvent manager, AS: auto sampler, TUV: tunable ultraviolet detector, BSM: binary solvent manager, MS: mass spectrometer, ACD: at-column-dilution. (**B**) Extracted drug species were transferred in a 5.50 min elution window using at-column-dilution with both valves in position 2 to refocus eluting drug species at the head of the analytical column. The transfer was bracketed with a 0.6 second interval in position 2,1 to purge the fluidic path.

**Figure 5.**
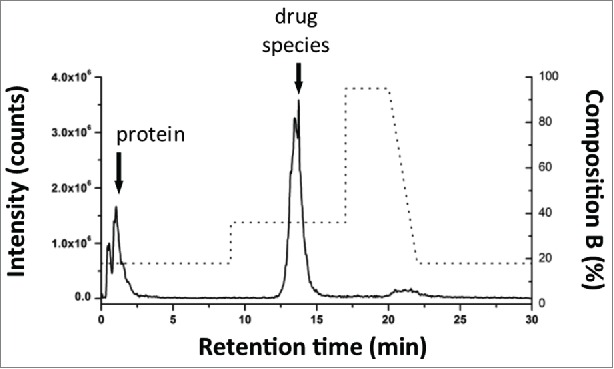
Method evaluation of SPE with spiked sample. Mal-linker-DSEA, and NAc-linker-DSEA was spiked into a dilute trastuzumab sample for SPE optimization. Optimal SPE loading conditions for the extraction of mal-linker-DSEA and NAc-linker-DSEA components from the spiked AFC sample were determined to be 18% acetonitrile containing 2% FA v/v. A step gradient to 36% acetonitrile containing 2% FA v/v was determined to be optimal conditions to elute bound drug components in a narrow peak centered around 13.5 min.

Method development for the proposed multidimensional approach started from the independent optimization of each dimension prior to coupling columns for AFC analysis. Optimization of solid phase extraction on the chosen SPE column was performed using a 2.1 × 20 mm, 30 um SPE column (Oasis MAX, Waters) to obtain the elution window required to transfer the mal-linker-DSEA and NAc-linker-DSEA from the 1^st^ dimension (SPE column) to the 2^nd^ dimension (analytical C_18_ column). For this purpose, we used the 2DLC configuration shown in Fig. 4A with a stainless steel union in lieu of the 2^nd^ dimension column (see Material and Methods section) and the left and right valve set in position 2. A small aliquot of the diluted trastuzumab sample was spiked with mal-linker-DSEA and NAc-linker-DSEA reference standards. The spiked trastuzumab sample was injected onto the SPE column with initial MP screening conditions based on RPLC retention times of the reference standards (see Material and Methods section). The loading and eluting MP composition was adjusted in an iterative fashion until no observable ions related to the reference standards were detected between the 5 min to 12 min and 15 min to 20 min portion of the MS chromatogram. The presence of ions between 5-12 min would indicate poor retention of reference standards, while ions detected between 15-20 min would indicate poor recovery of the reference standards. The optimized composition was determined to be 18% and 36% organic solvent for the loading and elution conditions, respectively. As shown in Fig. 5, under these conditions the protein molecules flow through to waste with the retained reference standards eluting in a relatively narrow window using a minimum amount of organic MP for improved at-column-dilution efficiency.

**Figure 6.**
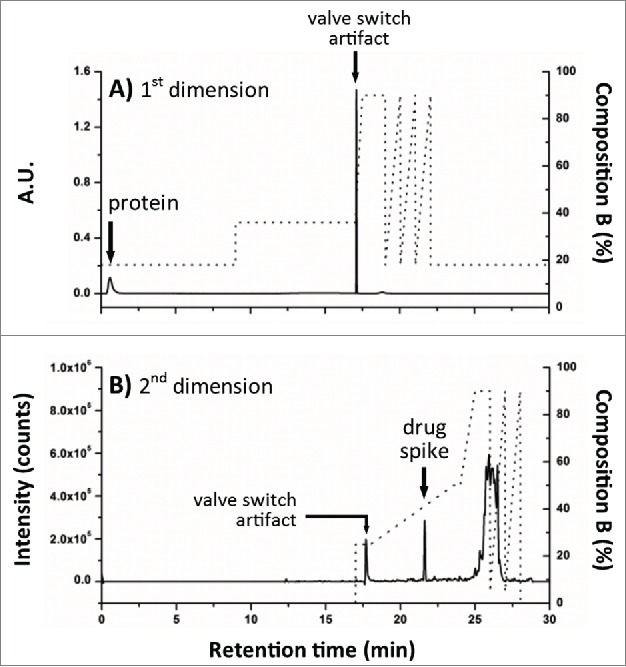
Evaluation of 2DLC configuration. NAc-linker-DSEA, spiked into a dilute trastuzumab sample, was successfully transferred from (**A**) the SPE column (1^st^ dimension) to the (**B**) RP column (2^nd^ dimension) using the 2DLC configuration illustrated in **Figure 4**A as proof-of-principle.

To verify the acquired experimental conditions in a real application setting, the stainless steel union was replaced with the 2^nd^ dimension superficially porous C_18_ RP column and initial valve states were set to position 1. A fresh aliquot of the diluted trastuzumab sample was spiked with just the NAc-linker-DSEA reference standard and injected using the optimized conditions as described for the AFC analysis with timed valve changes occurring as indicated in Fig. 4B. As shown in Fig. 6A, the protein was eluted in the void volume upon initial injection, allowing the spiked NAc-linker-DSEA to be successfully transferred and re-focused onto the 2^nd^ dimension RPLC column with subsequent elution using a 10 minute gradient (Fig. 6B). The independent control over mobile phase composition in the 1^st^ dimension and dilution ratio offers a higher degree of method control when optimizing for a variety of drug components that may exhibit different hydrophobic characteristics.^36^ The degree to which the eluent needs to be diluted will depend on the organic strength necessary to elute drug products from the 1^st^ dimension, but is readily adjusted using the proposed configuration.

### Recovery efficiency evaluation

Upon the successful development of the retention and transfer method for the NAc-linker-DSEA reference standard using the proposed 2DLC configuration, a natural extension is to test the recovery efficiency of the method. Four samples of the NAc-linker-DSEA were prepared at nominal concentrations of 2 ng/mL, 17 ng/mL, 74 ng/mL, 146 ng/mL, representing 4 points spanning the dynamic range of the method as determined by regression analysis of the quadrupole MS data. Injections were performed in triplicate in both 1DLC and 2DLC instrument configurations. Peak areas were compared for both instrument configurations to calculate the recovery. Recovery was determined to be within recommended guidelines,^56^ as shown in Table 2, with precision (R.SD) below 5% for both configurations and accuracy (R.E.) within 5%. These results indicate the SPE column was efficiently extracting the NAc-linker-DSEA sample from the eluent with no observable loss of standard in the void volume. In addition, the agreement in peak areas between both 1D and 2D configurations indicates transfer efficiency between columns was nearly 100% with no obvious loss that may be caused by the stainless steel tee used for at-column-dilution. The combined recovery results indicate the proposed multidimensional method is sufficiently robust and fit-for-purpose to deliver the recovery rate for the NAc-linker-DSEA standard across the established dynamic range.

**Table 2.** 2DLC recovery evaluation. Nac-linker-DSEA reference standard was prepared at 4 concentrations throughout the experimentally determined dynamic range and evaluate for recovery efficiency using the 2DLC configuration illustrated in **Figure 4**A. Identical separations were performed in a 1DLC configuration using the same system with a union in lieu of the SPE column (1^st^ dimension) as a reference. Comparison of peak area across triplicate injections of the NAc-linker-DSEA reference standard indicate sample recovery was equivalent between both 1DLC and 2DLC configurations.

Conc. (ng/mL)	1DLC			2DLC		
	Area	R.SD (%)	R.E. (%)	Area	R.SD (%)	R.E. (%)
2	458	5.2	95.4	482	1.7	100.4
17	4117	0.2	99.6	4146	1.0	100.3
74	17776	1.7	99.2	18063	2.7	100.8
146	35556	1.9	101.0	34839	0.2	99.0

### AFC sample analysis

**Figure 7.**
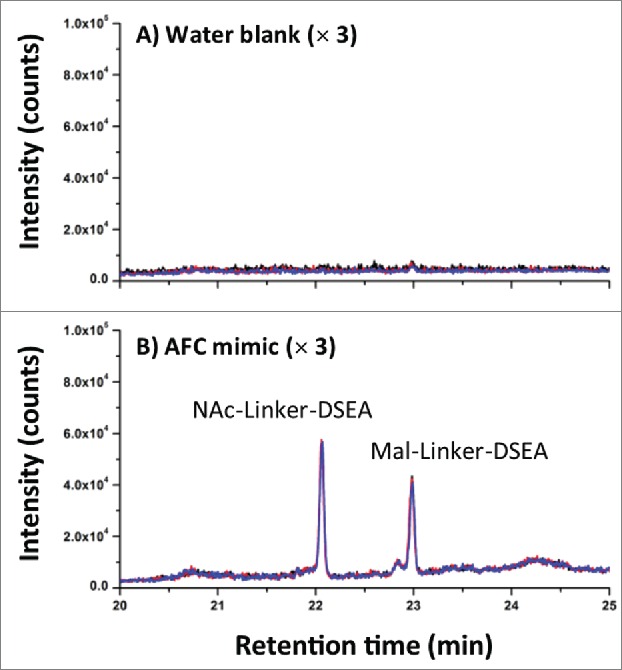
AFC sample chromatography. (**A**) Using the optimized 2DLC method as shown in **Figure 6**, a water blank was performed prior to each DSEA sample injection to monitor carry-over between DSEA sample injections. Overlay chromatograms of the 3 water blanks indicate no observable carry over of NAc-linker-DSEA and negligible carry over of mal-linker-DSEA (< 5% by area) between runs. (**B**) NAc-linker-DSEA and Mal-linker-DSEA drug components were detected in a 10 uL injection (19.4 ug) of neat AFC sample. Overlays of the 3 runs indicate a high degree of assay precision.

AFC samples were analyzed as received at a concentration of (1.94 mg/mL) without additional preparation for the detection of mal-linker-DSEA and NAc-linker-DSEA using the 2DLC configuration shown in Fig. 4A. A 10 μL injection of neat AFC sample was analyzed in triplicate using the same method as described above. The same experimental procedure was repeated using a water blank prior to each sample run to assess carry-over. As shown in Fig. 7A, triplicate SIR spectrum overlays of the blank injections show no observable carry-over of the NAc-linker-DSEA species at 22 min and negligible carry-over (< 5% by area) of the mal-linker-DSEA species eluting at 23 min indicating the method is reproducible and can be performed over multiple runs for extended column use. The presence of NAc-linker-DSEA and mal-linker-DSEA in the AFC sample was confirmed as shown in Fig. 7B with both species eluting reproducibly at 22 min and 23 min, respectively. Method precision was confirmed upon closer examination of the data as shown in Table 3, with R.SD determined to be less than 3% for both the mal-linker-DSEA and NAc-linker-DSEA drug components. Using the MS-based calibration plot (Fig. 3), the concentration of the free-drug components were determined to be 7.19 ng/mL and 3.82 ng/mL for the NAc-linker-DSEA and mal-linker-DSEA components, respectively. Detection of drug species at these levels, in a sample of modest concentration and injection volume, is not possible with optical detection alone, thus highlighting the utility of an MS detection in a 2DLC configuration such as this.

**Table 3.** AFC sample results. NAc-linker-DSEA and mal-linker-DSEA were detected at levels below UV detection thresholds in the AFC sample at concentrations of 7.19 ng/mL and 3.82 ng/mL, respectively.

Injection	NAc-Linker-DSEA			Mal-Linker-DSEA		
	Area	Experimental mass (pg)	Experimental conc. (ng/mL)	Area	Experimental mass (pg)	Experimental conc. (ng/mL)
1	3476	71.59	7.16	2716	38.67	3.87
2	3429	70.62	7.06	2746	39.10	3.91
3	3563	73.39	7.34	2599	36.98	3.70
**Mean**	3489	71.87	7.19	2687	38.25	3.82
**SD**	68	1.41	0.14	78	1.12	0.11
**R.SD**	1.95	1.96	1.96	2.89	2.92	2.92

## Discussion

Traditional methods for the detection, characterization, and quantification of trace free drug species in ADC samples have included assays based on ELISA and RPLC techniques with varying success.^19,23^ The reduction of sample preparation steps through the incorporation of multidimensional chromatography techniques has afforded analysts more efficient methods for assessment of trace drug species with improved sensitivity. Selection of orthogonal column chemistries for multi-dimensional assays can be a challenging prospect as eluents and format can affect assay specificity, efficiency, and sensitivity.^35-37^ The targeted removal of APIs from biological matrices using SPE-based techniques has been well established in the pharmaceutical industry. To that end, SPE techniques are becoming more prevalent in the characterization and routine testing of biopharmaceuticals.^22^ Method flexibility with off-line and on-line options combined with the ability to use volatile solvents makes SPE techniques ideal for multidimensional approaches with MS detection. The unique mixed mode OASIS chemistry facilitates the ability to separate complex mixtures based on different physicochemical properties and is ideal when considering the unique nature of ADCs that are composed of hydrophilic substrates conjugated to hydrophobic drugs.^57^ The current study has addressed challenges associated with residual free drug analysis through the development of a MS-compatible multidimensional approach that couples SPE-RPLC chemistries and is specific and sensitive.

Targeting free or non-conjugated hydrophobic drug species for selective extraction is achieved through the use of a hydrophobic SPE resin interspersed with anion exchange functional groups. Conceptually, net positively charged substrates such as mAbs or conjugated mAbs in solution at pH below their isoelectric point are passed through the positively charged SPE sorbent. In contrast, free drug and associated products are adsorbed to the mixed mode surface of the SPE ligand for downstream analysis. This study successfully demonstrated this approach through the capture and elution of a clinically relevant valine-citrulline-based surrogate molecule and its N-acetyl-cysteine quenched product from an unadulterated ADC mimic sample using an SPE-RPLC/MS approach. Similar hydrophobic characteristics observed in the surrogate molecules with established literature supports the viability of this method in clinical practice.^36^

The success of the proposed method relies on several key aspects working in a synergistic manner. The 1^st^ dimension is implementation of an SPE column that is retentive toward API species, which facilitates a means for analysts to tune the specificity of the method for varying substrates or drug candidates. In contrast, other 1^st^ dimension separations such as SEC do not provide the same degree of specificity or selectivity. In addition, the mixed mode SPE column efficiently removes protein substrates due to like-charge repulsion and reduces carry-over, allowing for repeated column use. Since trapped drug species are very hydrophobic, they are eluted at an organic percent that could reduce the 2^nd^ dimension RP chromatography performance. To overcome this challenge, at-column-dilution was incorporated to efficiently retain drug products in the 1^st^ dimension eluent at the head of the 2^nd^ dimension column.^22,58^ The degree to which the eluent needs to be diluted will depend on the organic strength necessary to elute drug products from the 1^st^ dimension, but is easily adjusted using the proposed configuration. The improved 2^nd^ dimension separation efficiency afforded by the inclusion of at-column-dilution, combined with in-line MS detection, increased the sensitivity of the assay 125-fold for the mal-linker-DSEA and 250-fold for the NAc-linker-DSEA drug species with a nominal LOQ of 0.3 ng/mL (1.5 pg on-column) compared to UV detection (Table 1). The current MS-based method represents over a 150-fold improvement in sensitivity compared to the SEC-RPLC/UV method described by Li *et al.* using similar, but not identical drug species.^36^ In addition to improved sensitivity, the ability to efficiently recover trace levels of drug species across a wide dynamic range makes the proposed method ideal for assessment of free drug species in formulation, stability studies, and clinical trials (e.g. biological matrices) associated with the production of ADCs.

As potentially more potent drug candidates for ADCs are identified,^33,34^ efforts to expand the therapeutic window will require assays with improved sensitivity for the assessment and characterization of residual free drug species to ensure product safety and efficacy.^57^ The utility of multidimensional approaches in the characterization of biopharmaceuticals is becoming increasingly evident.^35-39^ In this study, the SPE-RPLC/MS multidimensional approach combined with at-column-dilution was demonstrated as an efficient means to bypass lengthy sample preparation steps while enabling control over each dimension, promoting a method that can be readily adapted to existing workflows that is specific and sensitive for free drug analysis. Future work will include evaluation of the proposed method for extraction of free drug species using a diverse panel of clinically relevant ADCs in formulation and biological matrices.

## Material and methods

Chemicals and reagents were purchased from Sigma Aldrich unless otherwise stated. Mass spectrometry grade solvents were used for mobile phase and sample preparation.

### Antibody and linker-payload production and purification

This AFC is based on the conjugation of dansylsulfonamide ethyl amine (DSEAEA)-linker maleimide on interchain cysteines of trastuzumab used as a reference antibody. The trastuzumab used in this study is the European Union-approved version and formulation (21 mg/mL). The linker-fluorophore payload was designed to mimic the linker-drug most frequently used in ADC clinical trials. The synthesis was briefly reported in the supplemental material by Wagner-Rousset et al.^47^ It consists of maleimide-caproic acid dansylsulfonamide ethyl amine (mc_DSEA, structure Fig. 1A) with a valine-citruline linker that mimics the cytotoxic agent and linker conjugated to mAbs through reduced interchain cysteine via the maleimide function.

Mild reduction of trastuzumab and coupling of DSEA-linker were performed as previously described.^11^ Briefly, trastuzumab was reduced with 2.75 equivalents of TCEP in 10 mM borate pH 8.4 buffer containing 150 mM NaCl and 2 mM EDTA for 2 h at 37°C. The concentration of free thiols was determined by using the Ellman's reagent with L-cysteine as standard, typically resulting in around 5 thiols per antibody. To target a DAR of 4, the partially reduced trastuzumab was then alkylated with 2 equivalents of DSEA-linker per thiol in the same buffer for 1 h at room temperature. N-acetyl-cysteine (1.5 equivalents / DSEA- linker) was used to quench any unreacted DSEA-linker. The AFC was purified by SEC on a Superdex 200 pg column (GE Life Sciences) eluted with 25 mM histidine pH 6.5 buffer containing 150 mM NaCl, by using an AKTA Avant biochromatography system (GE Life Sciences). The AFC (average DAR = 4.0) was characterized by most of the methods used for hinge-Cys ADCs (nr/rSDS-PAGE, SEC, HIC, Native MS, LC-MS with IdeS/Red) and yielded similar profiles as those reported for brentuximab vedotin.^47,48^ Prepared AFC samples were used neat at a concentration of 1.94 mg/mL.

### Chromatography

An ACQUITY H-Class Bio equipped with a commercially available 2D technology configuration (Waters Corp.) was used for the experiments. Fig. 4A is a schematic of the instrument setup denoting column, pump, and plumbing configuration for 2DLC with at-column-dilution in place for 2^nd^ dimension loading. Transfer of retained analytes on the SPE column (1^st^ dimension) was performed through programmed valve events (Fig. 4B) using the column manager control interface. Valve switches were staggered with a 0.01 min delay to purge the at-column-dilution fluidic path prior to and after analyte transfer. A tunable UV detector (ACQUITY TUV, Waters Corp.) equipped with a 5-mm titanium flow cell was incorporated post 2^nd^dimension column to evaluate the optical detection limit of the separated analytes. Single wavelength detection was performed at an A_max_ of 280 nm with a sampling rate of 20 Hz. 1DLC experiments with the appropriate column and MP present in the 1^st^ dimension column position and quaternary solvent manager (QSM) reservoirs, respectively, were performed using the same system by physically interchanging fluidic path connections post column on both dimensions and leaving the 2^nd^ dimension Binary solvent manager (BSM) pump in an idle state with both valves in position 1.

### Column conditioning

A 2.1 × 20 mm, 30 μm SPE column (Oasis® MAX, Waters Corp.) was conditioned prior to sample runs using a dilute sample of trastuzumab (2 mg/mL) prepared in MS grade H_2_O with 0.1% FA v/v. Column conditioning was performed with the chromatography system in a 1DLC configuration at a flow rate of 0.300 mL/min with the column temperature set at 30 °C. QSM reservoirs were prepared as MP A: H_2_O, 2% FA v/v, MP B: acetonitrile, 2% FA v/v. A 2 μL injection of the conditioning sample was separated by performing a 10 min gradient from 0% MP B to 95% MP B until baseline line response stabilized. The 2^nd^ dimension column was conditioned in a similar fashion. A 2.1 × 50mm, 2.7 um superficially porous C_18_ column (Cortecs C_18_, Waters Corp.) was conditioned prior to actual sample runs using a dilute mixture of the reference standards (0.5 ng/mL) prepared in 50:50 ACN, 0.1% FA v/v : H_2_0, 0.1% FA v/v. Mobile phases were prepared as MP A: H_2_O, 0.1% FA v/v, MP B: acetonitrile, 0.1% FA v/v. A 5 μL injection of the diluted reference mixture was separated using a 10 min gradient from 5% MP B to 50% MP B at a flow rate of 0.300 ml/min and a column temperature of 40 °C. Injections were repeated until retention time and detector response stabilized for individual species. The system was re-plumbed in a 2DLC configuration for AFC analysis after conditioning of both columns.

### Calibration standards

Stock reference standards (Fig. 1) were dissolved in neat DMSO and prepared at concentrations of 4 μg/mL, 2.82 μg/mL, and 3.5 μg/mL for DSEA, mal-linker-DSEA, and NAc-linker-DSEA, respectively. Stock solutions were vortexed, briefly centrifuged, and divided into 50 μL aliquots and stored in -80°C prior to use. Individual stock reference solutions were diluted in a 1:5 ratio using 50:50 ACN 0.1% FA v/v: H_2_O 0.1% FA v/v to prepare initial calibration standard solutions. Sequential 1:1 serial dilutions were performed with the initial calibration standard solution for mal-linker-DSEA and NAc-linker-DSEA using 50:50 ACN 0.1% FA v/v : H_2_O 0.1% FA v/v. reference standards were evaluated using the chromatography system in a 1DLC configuration with the QSM reservoirs prepared as MP A: H_2_O, 0.1% FA v/v, MP B: acetonitrile, 0.1% FA v/v, MP C: and D: Acetonitrile. A 5.0 μL injection of each standard was loaded onto a 2.1 × 50mm, 2.7 um superficially porous C_18_ column (Cortecs C_18_, Waters) with the MP composition held constant for 1.0 min at 5% MP B at a flow rate of 0.300 ml/min and a column temperature of 40 °C. A 10 min gradient from 5% MP B to 50% MP B was used to elute the reference standard. Column reconditioning was performed using a rapid 1.0 min gradient to increase the organic composition to 80% MP B followed by a 1.0 min gradient to initial conditions (5% MP B) and held constant for 2 min.

### SPE optimization

Optimization of the 2.1 × 20 mm, 30 um SPE column (Oasis® MAX, Waters Corp.) was performed using a small aliquot of the dilute trastuzumab sample spiked with excess mal-linker-DSEA and NAc-linker-DSEA reference standards to increase MS response during acquisition. The LC instrument was configured in the 2DLC configuration shown in Fig. 4A using 2 6-port, 2-position valves housed in a column manager (ACQUITY column manager, Waters Corp.). For optimization purposes a stainless steel union was used in place of the 2^nd^ dimension column and both valves were set to an initial position of 2. The 1^st^ dimension QSM reservoirs were prepared as MP A: H_2_O, 2% FA v/v, MP B: acetonitrile, 2% FA v/v, MP C: and D: Acetonitrile. The 2^nd^ dimension BSM reservoirs were prepared as MP A: H_2_O, 0.1% FA v/v, MP B: acetonitrile, 0.1% FA v/v. The 2^nd^ dimension BSM was programmed to flow at a rate of 0.300 mL/min with a MP composition of 60% MP B (2^nd^ dimension MP reservoirs) to replicate back pressure on the 1^st^ dimension column encountered when both columns are in-line. The spiked trastuzumab sample was injected onto the SPE column at a flow rate of 0.100 ml/min with an initial MP composition of 23% MP B (1^st^ dimension MP reservoirs). The gradient was then stepped up to 54% MP B to elute the retained reference standards. The initial and eluting MP composition was adjusted in an iterative fashion until no observable ions related to the reference standards were detected between the 5 min to 12 min and 15 min to 20 min portion of the MS spectrum. The optimized composition was determined to be 18% MP B for the loading conditions and 36% MP B for the elution conditions. Once optimized, the stainless steel union was replaces with the 2^nd^ dimension superficially porous C_18_ column and initial valve states were set to position 1. Proof-of-principle was performed using a fresh aliquot of the dilute trastuzumab sample spiked with only the NAc-linker-DSEA reference standard and injected using the optimized conditions described in the AFC sample analysis.

### AFC sample analysis (1^st^ dimension)

AFC samples were analyzed using an optimized 2-step gradient in the 1^st^ dimension. QSM reservoirs were prepared as MP A: H_2_O containing 2% FA v/v, MP B: acetonitrile containing 2% FA v/v, MP C: and MP D: Acetonitrile. Neat AFC samples were injected at a volume of 10.0 μL using isocratic conditions set at 18% MP B at a flow rate of 0.100 ml/min and a column temperature of 30 °C. After 9 min the MP composition was stepped up to 36% MP B and held constant for 8 min to elute the bound analyte. Transfer of analytes to the 2^nd^ dimension RPLC column was achieved through a programmed valve event where the left and right valves were switched to position 2 between the 12.00 and 17.50 min mark of the gradient to combine the fluidic path of the 1^st^ and 2^nd^ dimension columns. A sharp 0.50 min gradient was used to increase the MP composition to 90% MP B from 17.0 to 17.5 min and held constant for an additional 2.50 min. A saw-tooth gradient from 90% MP B to 18% MP B was cycled 3 times to recondition the Oasis MAX SPE column with the final cycle returning to the initial start conditions.

### AFC sample analysis (2^nd^ dimension)

BSM reservoirs were prepared as MP A: H_2_O containing 0.1% formic acid v/v, MP B: acetonitrile containing 0.1% formic acid v/v. As part of the 2DLC method the 2^nd^ dimension MP composition was set at 0% MP B at the time of injection and held constant until the 17.50 min mark at a flow rate of 0.300 ml/min. At-column-dilution was performed in a 1:4 dilution (0.1 mL/min 1^st^ dimension pump: 0.3 mL/min 2^nd^ dimension pump) via a stainless steel tee (Vicci Valco) while the 1^st^ and 2^nd^ dimension fluidic paths were combined between 12.00 and 17.50 min of the method. After 17.50 min MP composition was stepped to 25% MP B and held for 1 min. A 5.55 min gradient was performed from 25% MP B to 50% MP B and held constant for an additional 0.44 min. After the separation gradient was performed the MP composition was ramped to 90% MP B in 1 min followed by two 1 min saw-tooth gradients from 90% MP B to 5% MP B to recondition the RPLC column with the final cycle returning to the initial start conditions.

### Recovery evaluation

Assessment of recovery efficiency of the SPE column was performed using the NAc-linker-DSEA reference standard. Four samples were prepared at concentrations of 1.9 ng/mL, 17.0 ng/mL, 74.0 ng/mL, and 145.5 ng/mL in 50:50 ACN 0.1% FA v/v: H_2_0 0.1% FA v/v to span the dynamic range based on the NAc-linker-DSEA calibration plot (Fig. 3). Using the 2DLC system configuration for the AFC sample analysis described earlier, Injections were performed in triplicate for 4 reference standard samples. The system was then re-configured in a 1DLC mode, and the QSM reservoirs were changed to MP A: H_2_O containing 0.1% FA v/v, MP B: Acetonitrile containing 0.1% FA v/v. Reservoir C: and D: were left idle with neat Acetonitrile. The same reference samples were directly injected onto the 2.1 × 50mm, 2.7 um superficially porous C_18_ column with the MP composition held constant for 1.0 min at 5% MP B at a flow rate of 0.300 ml/min and a column temperature of 40 °C. A 10 min gradient from 5% MP B to 50% MP B was used to elute the reference standard. Column reconditioning was performed using a rapid 1.0 min gradient to increase the organic composition to 80% MP B followed by a 1.0 min gradient to initial conditions 5% MP B and held constant for 2 min. Recovery efficiency was determined by comparing peak area in both 1DLC and 2DLC system configurations.

### MS Settings

A single quadrupole mass spectrometer (ACQUITY QDa, Waters Corp.) was used for MS analysis post TUV detection (Fig. 4A). SIRs representing the [M+1H]^+1^ and [M+2H]^+2^ of the DSEA, mal-linker-DSEA, and NAc-linker-DSEA were acquired in positive polarity covering a mass to charge range of 30 to 1,250 m/z. A confirmed fragment of the mal-linker-DSEA (m/z 718.4) was also acquired in addition to the other charge states and used for MS optimization. MS data was collected throughout the separation as defined in the chromatography section with the flow continuously passing through the MS capillary. Adjustable instrument settings were set as follows: capillary voltage 0.8 kV, sample cone 2.0 V, source temperature 400 °C. Data from the MS analysis were processed within the chromatography data system MassLynx. Respective SIRs for the DSEA, mal-linker-DSEA, and NAc-linker-DSEA samples were summed and a mean smoothed applied with a window size of 5 scans and 1 iteration, followed by integration.
